# 2019 JBSR Report

**DOI:** 10.5334/jbsr.2107

**Published:** 2020-06-15

**Authors:** Alain Nchimi

**Affiliations:** 1 CHL, LU

The *Journal of the Belgian Society of Radiology* (JBSR) is now in its fifth year as a free access online journal. During the tenure of the current editor-in-chief, the only print exceptions have been related to a special scientific event, such as the annual meetings of the Belgian Society of Radiology (BSR). In this report we briefly describe noticeable evolutions of the journal across this time with emphasis on the past year.

## Journal Content

The journal content has progressively shifted towards a preference for state-of-the-art and original paper articles over case reports. Nevertheless, the JBSR is strongly committed to encouraging Belgian radiologists’ submissions, as these are often among the authors’ first scientific articles. For this reason, high-quality case reports and images in clinical radiology articles with great teaching values are still being considered, though they represent a decreasing proportion of articles published every year.

## Audience

**Figure 1:**
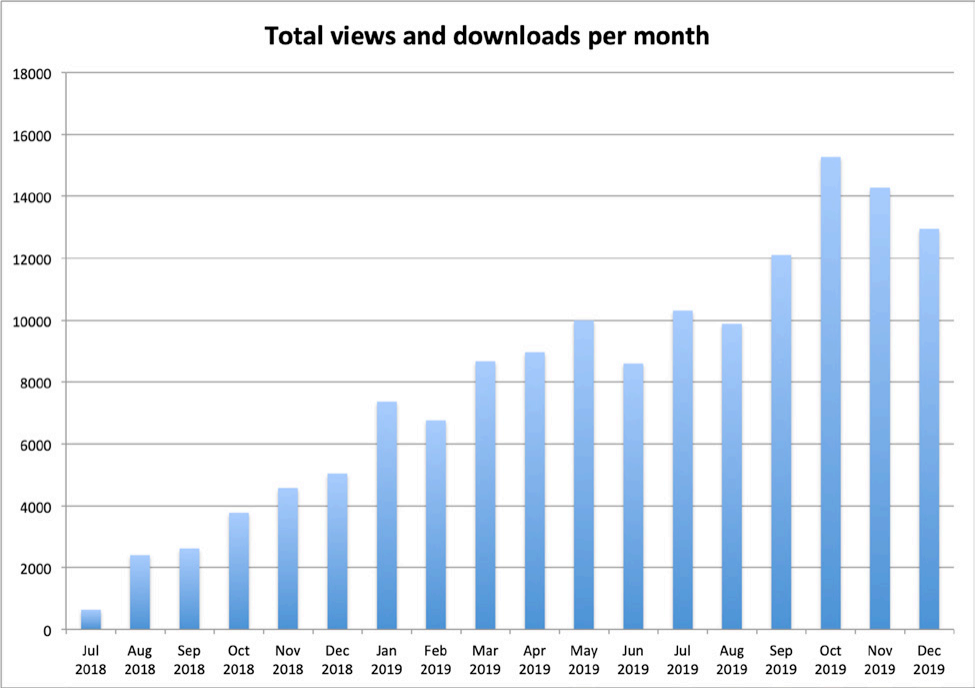
Combined JBSR article views and downloads per month in 2018–2019.

Sustaining the journal content trends over the last five years did little to prevent the JBSR from keeping a strong national identity. In 2019, the number of submissions from Belgium-based authors equalled the sum of contributions from all other countries, with Turkey and South Korea being respectively ranked second and third. In contrast, the journal is fully open to the world’s scientific community, and Belgium ranks only eighth among countries from which the number of article views and downloads originate (Table 1). We are thankful that a strong increase in article views and downloads has been another general trend for the JBSR in 2019, both through PubMed Central (Figure 1) and the journal’s website. The latter peaked at the end of the year, around the date of the annual meeting of the Belgian Society of Radiology.

**Table 1:** Submission and session views for the JBSR in 2019.

Top 10 submissions by Country in 2019	Number

Belgium	114
Turkey	65
Republic of Korea	33
India	20
Republic of China + Taiwan + Hong Kong	18
Japan	8
United Kingdom	6
United States of America	5
Saudi Arabia	4
France	4
**Top 10 session views and downloads per country in 2019**	

United States of America	128,835
India	38,721
United Kingdom	18,386
Canada	10,610
Australia	10,400
Republic of Korea	9,855
Belgium	7,582
Turkey	7,085
Brasil	6,300
Philippines	4,119

## Editorial Decisions

The submission acceptance rate dropped from 35% in 2018 to 23% in 2019, due to a higher editorial selection process. As a result, the 2018 JBSR impact factor reached 0.475, while it was only 0.027 in 2017. Despite the journal’s ongoing success, articles’ turnaround time remain high. The timelines for submission-to-acceptance or -to-rejection and acceptance-to-publication were respectively 88, 55, and 83 days. Similar to many biomedical journals, the JBSR faces difficulties regarding external peer reviewing, which delays editorial decisions. Expediting our decisions will force us to re-think the peer-reviewing strategy of the journal, asking for more participation from the authors in the choice of available peer reviewers.

